# Reproducibility of the Ribosomal RNA Synthesis Ratio in Sputum and Association with Markers of Mycobacterium tuberculosis Burden

**DOI:** 10.1128/Spectrum.00481-21

**Published:** 2021-09-08

**Authors:** Emmanuel Musisi, Christian Dide-Agossou, Reem Al Mubarak, Karen Rossmassler, Abdul Wahab Ssesolo, Sylvia Kaswabuli, Patrick Byanyima, Ingvar Sanyu, Josephine Zawedde, William Worodria, Martin I. Voskuil, Rada M. Savic, Payam Nahid, J. Lucian Davis, Laurence Huang, Camille M. Moore, Nicholas D. Walter

**Affiliations:** a Infectious Disease Research Collaboration, Kampala, Uganda; b Department of Biochemistry, Makerere University, Kampala, Uganda; c Department of Medical and Biological Sciences, Infection and Global Health Division, University of St. Andrews, St. Andrews, United Kingdom; d Department of Epidemiology, Colorado School of Public Health, Aurora, Colorado, USA; e Rocky Mountain Regional VA Medical Center, Aurora, Colorado, USA; f Division of Pulmonary Sciences and Critical Care Medicine, University of Colorado Anschutz Medical Campus, Aurora, Colorado, USA; g Department of Immunology and Microbiology, University of Colorado Anschutz Medical Campus, Aurora, Colorado, USA; h Consortium for Applied Microbial Metrics, Aurora, Colorado, USA; i Department of Bioengineering and Therapeutic Sciences, University of California San Francisco, San Francisco, California, USA; j Division of Pulmonary and Critical Care Medicine, University of California San Francisco, San Francisco, California, USA; k Division of HIV, Infectious Diseases, and Global Medicine, University of California San Francisco, San Francisco, California, USA; l UCSF Center for Tuberculosis, San Francisco, California, USA; m Department of Epidemiology of Microbial Diseases, Yale School of Public Health, New Haven, Connecticut, USA; n Pulmonary, Critical Care, and Sleep Medicine Section, Yale School of Medicine, New Haven, Connecticut, USA; o Zuckerberg San Francisco General Hospital, San Francisco, California, USA; p Division of Biostatistics and Bioinformatics, National Jewish Health, Denver, Colorado, USA; University of Arizona/Banner Health

**Keywords:** human, *Mycobacterium tuberculosis*, sputum, assay development, pharmacodynamics

## Abstract

There is a critical need for improved pharmacodynamic markers for use in human tuberculosis (TB) drug trials. Pharmacodynamic monitoring in TB has conventionally used culture or molecular methods to enumerate the burden of Mycobacterium tuberculosis organisms in sputum. A recently proposed assay called the rRNA synthesis (RS) ratio measures a fundamentally novel property, how drugs impact ongoing bacterial rRNA synthesis. Here, we evaluated RS ratio as a potential pharmacodynamic monitoring tool by testing pretreatment sputa from 38 Ugandan adults with drug-susceptible pulmonary TB. We quantified the RS ratio in paired pretreatment sputa and evaluated the relationship between the RS ratio and microbiologic and molecular markers of M. tuberculosis burden. We found that the RS ratio was highly repeatable and reproducible in sputum samples. The RS ratio was independent of M. tuberculosis burden, confirming that it measures a distinct new property. In contrast, markers of M. tuberculosis burden were strongly associated with each other. These results indicate that the RS ratio is repeatable and reproducible and provides a distinct type of information from markers of M. tuberculosis burden.

**IMPORTANCE** This study takes a major next step toward practical application of a novel pharmacodynamic marker that we believe will have transformative implications for tuberculosis. This article follows our recent report in *Nature Communications* that an assay called the rRNA synthesis (RS) ratio indicates the treatment-shortening of drugs and regimens. Distinct from traditional measures of bacterial burden, the RS ratio measures a fundamentally novel property, how drugs impact ongoing bacterial rRNA synthesis.

## INTRODUCTION

To achieve the World Health Organization End TB strategic goals, it will be necessary to develop new, shorter treatment regimens for both drug-susceptible and drug-resistant tuberculosis (TB) (1, 2). One key challenge for evaluation of new TB treatment regimens is the limitation of existing pharmacodynamic (PD) markers (3–5). There is an urgent need for new PD markers that maximize information gained from preclinical animal models and early-phase human clinical trials. More accurate PD markers would enable selection of the most efficacious regimens for testing in definitive phase III trials (6–8).

**FIG 1:**
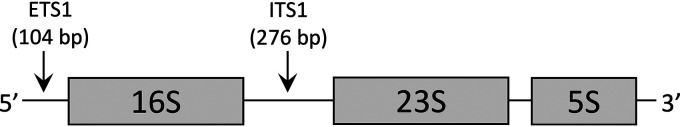
Schematic of ribosomal operon in M. tuberculosis, illustrating rapidly degraded precursor spacer sequences (ETS1 and ITS1). The RS ratio is based on the abundance of ETS1 sequence relative to 23S rRNA sequence.

We recently proposed a new marker of TB treatment efficacy called the rRNA synthesis (RS) ratio (9). In Mycobacterium tuberculosis, the three rRNA sequences are transcribed on a single polycistronic precursor-rRNA (pre-rRNA) sequence with intervening short spacer sequences called internally transcribed spacer 1 (ITS1) and externally transcribed spacer 1 (ETS1) (Fig. 1). Since the spacer sequences are rapidly degraded, they serve as a marker of newly transcribed rRNA (10). The RS ratio estimates ongoing rRNA synthesis in M. tuberculosis populations by quantifying the relative abundance of ETS1 sequence relative to 23S rRNA sequence.

The RS ratio is unlike most conventional and investigational PD markers that enumerate the burden of M. tuberculosis, such as sputum smear grade, time to positivity (TTP) in liquid culture, GeneXpert MTB/RIF (Xpert) threshold cycle (*C_T_*) values, and M. tuberculosis rRNA burden. In contrast, the RS ratio measures the effect of drugs on the physiologic state of the pathogen. In principle, the RS ratio provides a different type of information than these existing measures of M. tuberculosis burden. Key insights from *in vitro* and murine studies are (i) drugs often affect the RS ratio and CFU burden differently and (ii) the RS ratio appears to indicate the sterilizing activity of drugs and regimens (9). This novel molecular approach has yet to be extensively investigated as a marker in humans.

As a preliminary evaluation of the performance of the RS ratio in human sputum, we evaluated pretreatment sputa from 38 Ugandan adults with drug-susceptible pulmonary TB. We tested the repeatability and reproducibility of the RS ratio in paired pretreatment sputa and evaluated the association of the RS ratio with conventional and investigational PD markers that enumerate M. tuberculosis burden. Our results suggest that the RS ratio is repeatable and reproducible and provide a distinct new type of information.

## RESULTS

### Study population characteristics.

Evaluation of 102 adults confirmed drug-susceptible pulmonary TB among 52 participants. After excluding 14 who declined to participate or were unable to produce additional sputa, 38 participants were included in this study (see Fig. S1 in the supplemental material). Table 1 provides participant characteristics.

**TABLE 1:** Participant characteristics^*a*^

Variable	Value
Age in yrs, median (IQR)	34 (26–38)
Wt in kg, median (IQR)	53 (50–59)
Female (%)	32
HIV-uninfected (%)	66
Nonsmoking (%)	82
Smear grade (%)	
Negative	3
Positive 1+	32
Positive 2+	32
Positive 3+	26
Other	8
TTP in days, median (IQR)	6 (4–9)
Xpert *C_T_* cycles, median (IQR)	18.2 (16.3–20.0)
16S burden, median (IQR)^*b*^	
SS1	6.0 (4.9–7.6)
SS2	5.8 (4.8–7.3)
23S burden, median (IQR)^*b*^	
SS1	5.4 (4.1–7.3)
SS2	5.2 (3.9–6.7)
RS ratio, median (IQR)^*b*^	
SS1	3.3 (3.0–3.5)
SS2	3.3 (3.2–3.6)

a IQR, interquartile range; *C_T_*, cycle threshold; TTP, time to culture positivity.

b log_10_-transformed.

### Repeatability and reproducibility of RS ratio in sputum.

In the paired pretreatment sputa, the RS ratio was quantifiable in 97% of first sputum samples (SS1) and 92% of second sputum samples (SS2). When SS1 samples were assayed in triplicate in a single experiment for assessment of repeatability, the intraclass correlation coefficient (ICC) was 0.99. When the RS ratio was conducted by two different lab workers using different instruments at a 12-month interval, interobserver reproducibility was high (ICC, 0.93). Repeatability and reproducibility results are summarized in Table 2.

**TABLE 2:** Repeatability and reproducibility of the RS ratio in sputum

Measure	Sample	Basis for assessment	Intraclass correlation coefficient
Repeatability in technical replicates^*a*^	SS1	Same sputum sample Same operator Same expt	0.99 (0.99–1.00)
Interobserver reproducibility^*b*^	SS1	Same sputum sample Different operators Different instruments Different experiments	0.93 (0.86–0.97)
Biological reproducibility^*c*^	SS1 vs SS2	Paired sputum samples Same operator Same instrument Same expt	0.63 (0.37–0.79)

a Agreement among three replicate RS ratio results within a single experiment.

b Agreement between RS ratio results in the same samples conducted by two different lab workers using different instruments at a 12-month interval.

c Agreement between RS ratio results in two separate sputum samples collected within an hour of each other.

When the RS ratio was compared between two paired sputum samples from the same participant, the ICC was 0.63, indicating good biological reproducibility. The RS ratio estimates did not differ systematically between the first and second sputum sample (mean difference between RS ratios in SS1 and SS2, –0.11 [95% confidence interval (CI), –0.26 to 0.03]). The variability of the RS ratio between paired sputum samples (ICC, 0.63) was comparable to the variation observed in paired sputum samples in M. tuberculosis 16S rRNA (ICC, 0.64) and 23S rRNA (ICC, 0.64).

### Association of RS ratio with markers of M. tuberculosis burden.

There was no significant relationship between RS ratios quantified in SS1 and M. tuberculosis rRNA burden, smear grade, TTP, or Xpert *C_T_* value (Table 3), reinforcing our understanding that the RS ratio provides a different type of information than markers of M. tuberculosis burden. Similar results were observed using RS ratios quantified in SS2 (Table 3). Conversely, with few exceptions, M. tuberculosis rRNA burden, smear grade, TTP, and Xpert *C_T_* values were significantly associated, despite the fact that some measurements were made on different sputum samples.

**TABLE 3:** Association between the RS ratio in SS1 and SS2 and markers of M. tuberculosis burden^*a*^*^,^*^*d*^

Markers	RS ratio^*b*^	16S rRNA burden^*b*^	23S rRNA burden^*b*^	Smear grade	TTP in liquid culture	Xpert *C_T_* values
SS1						
RS ratio^*b*^		*ρ* = –0.19 (*P* = 0.28)	*ρ* = –0.20 (*P* = 0.24)	*ρ* = –0.15 (*P* = 0.40)	*ρ* = 0.22 (*P* = 0.21)	*ρ* = 0.43 (*P* = 0.08)
16S rRNA burden^*b*^			*ρ* = 0.98 (*P* < 0.01)	*ρ* = 0.49 (*P* < 0.01)	*ρ* = –0.64 (*P* < 0.01)	*ρ* = –0.65 (*P* < 0.01)
23S rRNA burden^*b*^				*ρ* = 0.45 (*P* < 0.01)	*ρ* = –0.57 (*P* < 0.01)	*ρ* = –0.59 (*P* = 0.01)
Smear grade					*ρ* = –0.72 (*P* < 0.01)	*ρ* = –0.07 (*P* = 0.80)
TTP in liquid culture						*ρ* = 0.62
						(*P* < 0.01)
Markers	**RS ratio** ^***c***^	**16S rRNA burden** ^***c***^	**23S rRNA burden** ^***c***^	**Smear grade**	**TTP in liquid culture**	**Xpert** ***C_T_*** **values**
SS2						
RS ratio^*c*^		*ρ* = –0.05 (*P* = 0.75)	*ρ* = –0.04 (*P* = 0.83)	*ρ* = 0.12 (*P* = 0.50)	*ρ* = –0.08 (*P* = 0.65)	*ρ* = 0.10 (*P* = 0.68)
16S rRNA burden^*c*^			*ρ* = 0.97 (*P* < 0.01)	*ρ* = 0.48 (*P* < 0.01)	*ρ* = −0.50 (*P* < 0.01)	*ρ* = –0.37 (*P* = 0.13)
23S rRNA burden^*c*^				*ρ* = 0.52 (*P* < 0.01)	*ρ* = –0.50 (*P* < 0.01)	*ρ* = –0.38 (*P* = 0.12)

a Spearman correlation coefficients (*ρ*) with *P* values (*P*) are provided. RS ratio and rRNA burden measurements were log_10_-transformed. *C_T_*, cycle threshold; TTP, time to culture positivity.

b Using RS ratio, 16S and 23S rRNA from SS1.

c Using RS ratio, 16S and 23S rRNA from SS2.

d Sputum grade was converted to an ordinal scale with negative, positive 1+, positive 2+, and positive 3+ corresponding to 0, 1, 2, and 3, respectively.

### Association of clinical and demographic factors with the RS ratio and markers of M. tuberculosis burden.

In general, there was no significant relationship between clinical and demographic factors, including sex, HIV status, and smoking, and the RS ratio or markers of M. tuberculosis burden. The only exception was for the relationship between Xpert *C_T_* values and sex, which had a *P* value of 0.03 (Table S1). After adjustment for multiple comparisons, this association was nonsignificant.

## DISCUSSION

Using sputa from Ugandan adults with untreated TB, we evaluated the RS ratio in pretreatment sputa and compared the RS ratio with other conventional and investigational markers. We found that the RS ratio was highly repeatable in technical replicates and had high interobserver reproducibility. The sputum-to-sputum biological variability in the RS ratio approximated the variability observed in M. tuberculosis 16S and 23S rRNA burden. Comparison of the RS ratio with sputum smear, TTP, Xpert *C_T_* values and M. tuberculosis rRNA burden indicated that the RS ratio measures a distinct property that is independent of bacterial burden.

An important precursor to establishing a novel PD marker is understanding technical and biological variability of the assay (11–13). The sputum RS ratio demonstrated high technical consistency with high repeatability and interobserver reproducibility, consistent with the previously described performance of droplet digital PCR (ddPCR) in clinical samples (14, 15).

As anticipated, the biological variability exceeded the technical variability. The baseline sample is particularly important because clinical trials typically evaluate how changes in PD marker over time relative to baseline values relate to disease outcomes. Therefore, evaluating the repeatability and reproducibility of the RS ratio in paired baseline sputum samples helps us understand the source of variability. We suspect that the sources of biological variability differ between the RS ratio and markers that enumerate M. tuberculosis burden. Generally, production of greater sputum volume is associated with higher M. tuberculosis burden and correspondingly shorter TTP, lower Xpert values, and higher M. tuberculosis rRNA burden (16–18). The volume of sputum varies based on time of collection, participant effort to expectorate, and severity of lung disease (16). In contrast, the RS ratio is designed to be “self-normalizing” to bacterial burden because both the pre-rRNA numerator and the 23S rRNA denominator scale with change in M. tuberculosis burden. For the RS ratio, it is likely that sputum-to-sputum variation indicates biological variability in the M. tuberculosis populations present in samples originating from different regions of the lung (19, 20).

Consistent with our hypothesis that the RS ratio is not a marker of M. tuberculosis burden, we did not observe an association between this novel marker and conventional or investigational markers that enumerate M. tuberculosis burden. This is similar to findings from our *in vitro* and murine studies (9). Our findings suggest that the RS ratio is a feasible tool for evaluating human sputa that may complement markers of M. tuberculosis burden, providing novel insight into treatment response.

This study has several limitations. First, although our goal is a PD marker that can be used to monitor treatment effectiveness, here, we studied only pretreatment samples. Nevertheless, evaluation of technical and biological variability in pretreatment samples is an important preliminary step required for interpretation of longitudinal data. Second, by necessity, we used different sputum samples to evaluate different markers. For example, smear status and TTP were quantified in one sample, Xpert was quantified in another, and RS ratio and M. tuberculosis rRNA burden were quantified in separate paired samples. Nonetheless, all of the markers that enumerate M. tuberculosis burden were strongly associated.

In summary, this study determined that assaying the RS ratio in sputum is both repeatable and reproducible. The RS ratio may serve as a novel PD marker that offers a new physiologic perspective on TB treatment, distinct from existing assays of M. tuberculosis burden.

## MATERIALS AND METHODS

### Participant recruitment and specimen collection.

Participants were enrolled at Naguru Referral Hospital in Kampala, Uganda, from August 2018 to March 2019 as a component of a longitudinal observational cohort study of adults with pneumonia called the International HIV-Associated Opportunistic Pneumonias-Inflammation, Ageing, Microbes, and Obstructive Lung Disease study (21, 22). Participants were ≥18 years of age, with persistent cough, without signs of extrapulmonary TB, and without TB treatment within the past 2 years.

Each participant provided 4 spot sputum samples. The first and second sputum samples were processed for smear microscopy, liquid and solid cultures, and Xpert, as described in the supplemental material. Auramine O fluorescent smear microscopy used the direct method. Drug-susceptible pulmonary TB was confirmed by sputum smear microscopy, culture, and Xpert (23). Smear microscopy used a small amount of primary sputum. Culture and Xpert each used 1 ml of processed sputum. Two additional sputum samples were collected within a 1-h time interval for RNA-based assays in a guanidine thiocyanate (GTC)-based RNA preservative as described in the supplemental material. Both the parent and current study were approved by the institutional review boards in Uganda and the United States. All participants provided written informed consent for the use of their sputa and clinical data for a biomarker study.

### RNA extraction and quantification of rRNA burden and RS ratio.

Total RNA was extracted from paired sputum samples using standard methods described in the supplemental material. Following reverse transcription, 16S and 23S rRNA transcripts were quantified in triplicate via reverse transcription quantitative PCR (RT-qPCR). Absolute copies were determined by reference to a standard DNA ladder. Employing methods similar to those used for the molecular bacterial load assay (24, 25), we used a spike-in to adjust for loss in RNA extraction (“retention percentage”) and normalized by sputum weight to estimate the burden of 16S or 23S rRNA in sputum (supplemental material). For the RS ratio assay, droplet digital PCR (ddPCR) was used to quantify the abundance of pre-rRNA relative to 23S rRNA, as previously described (9).

### Evaluation of repeatability and reproducibility.

To understand the sources of variability in the RS ratio, we defined repeatability and reproducibility in three ways. Repeatability was the agreement among three replicate RS ratio results within a single experiment (i.e., technical replicates). Second, interobserver reproducibility was the agreement between RS ratio results in the same samples conducted by two different lab workers using different instruments at a 12-month interval. Finally, we defined a sputum-to-sputum biological reproducibility as the agreement between RS ratio results in two separate sputa (SS1 and SS2) collected within an hour of each other. We additionally evaluated variability in measurement of M. tuberculosis rRNA burden by qPCR, quantifying repeatability in technical replicates and sputum-to-sputum biological reproducibility. Repeatability and reproducibility were estimated based on the intraclass correlation coefficient (ICC), ranging from 0, indicating no agreement, to 1, indicating perfect agreement (26). We implemented the one-way random effects ICC framework (27), which assumes that each participant is measured by a different set of assays, using the function icc in the irr R package.

### Comparison of RS ratio with existing and investigational markers of M. tuberculosis burden.

We tested the association of the RS ratio with sputum smear grade, TTP, Xpert, and rRNA burden using Spearman correlation tests. For the burden of 16S and 23S rRNA, we selected the median of triplicates for statistical analysis. *P* values of <0.05 were considered statistically significant. Relationships of the RS ratio and markers of M. tuberculosis burden with clinical and demographic factors were tested using two-sample Wilcoxon tests. Statistical analysis was conducted in R v 3.5.3 (R Development Core Team, Vienna, Austria).

### Data availability.

All primary data are included in the supplemental material.
