# Supplementary material for: Reproducibility of the Ribosomal RNA Synthesis Ratio in Sputum and Association with Markers of Mycobacterium tuberculosis Burden
Source: Microbiol Spectr. 2021 Sep 8;9(2):e00481-21. doi: 10.1128/Spectrum.00481-21 (PMC8557932; doi:10.1128/Spectrum.00481-21)
Supplement: SUPPLEMENTAL FILE 1 — Supplemental material. Download SPECTRUM00481-21_Supp_1_seq1.pdf, PDF file, 0.3 MB [file spectrum00481-21_supp_1_seq1.pdf]

SUPPLEMENTAL MATERIAL

Reproducibility of the ribosomal RNA synthesis ratio in sputum and association with markers of *M. tuberculosis* burden

Emmanuel Musisi,[1,2,3*†] Christian Dide-Agossou,[4*†] Reem Al Mubarak,[5,6] Karen Rossmassler,[5,6] Abdul Wahab Ssesolo,[1] Sylvia Kaswabuli,[1] Patrick Byanyima,[1] Ingvar Sanyu,[1] Josephine Zawedde,[1] William Worodria,[1] Martin I Voskuil,[7,8] Rada M Savic,[8,9,10,11] Payam Nahid,[8,10,11,12] J. Lucian Davis,[13] Laurence Huang,[10,11,14] Camille M Moore,[15] Nicholas D Walter[5,6,8]

***** These authors contributed equally to this work (EM, CD-A)
† Corresponding authors (EM, CD-A)

Table of Contents

Supplemental methods 2
Bacteriological assays 2
Sputum processing for RNA assays 3
RNA extraction 4
Quantification of rRNA burden via RT-qPCR 4
Quantification of RS Ratio via droplet digital PCR 5
Supplemental figures & tables 6
FIG S1. Participant recruitment and sputum collection. 6
FIG S2. Graphical representation of results presented in Table 3. 7
TABLE S1. Associations of gender, HIV status and smoking with RS Ratio and markers of Mtb burden. 8
References 9

SUPPLEMENTAL METHODS

Bacteriological assays

Smear microscopy

Auramine-O- fluorescent smear microscopy was done using direct method. A small amount of primary sputum was placed on a slide for the purpose of microscopic examination. Dried smears (1–2 cm) were stained for 15 mins using a 0.5% solution of auramine-O (Merck, Darmstadt, Germany), decolorized for 2 mins in 3% acid alcohol, and counterstained for 1 min in 0.5% potassium permanganate solution. Smears were examined within 1h under a fluorescent microscope at 400 magnification. Sputum smear results were graded (negative, scanty, 1+, 2+, 3+) according to the International Union Against Tuberculosis and Lung Disease guidelines (1).

Liquid and solid cultures

Sputum was homogenized mechanically by vortexing the sample with sterile beads. Decontamination was achieved by treating the homogenized sputum with equal volume of NaOH/N-acetyl L-cysteine (NALC) (*i.e.*, fresh 2% solution prepared with 2.9% trisodium citrate and 0.5 g NALC), neutralized with sterile PBS, pH 6.8, and centrifuged at 3000g for 20 min and the resulting pellet was resuspended in 2 ml of phosphate buffered saline before inoculating 1ml into the MGIT and solid culture media, respectively. Maximum incubation time for liquid culture (BACTEC MGIT 960 System; Becton Dickinson Franklin Lakes, NJ, USA) was 42 days. Results and time to culture positivity (TTP) were both instrument-determined. Positive MGIT tubes were cultured on blood agar to assess for contaminating bacteria. For solid culture on Lowenstein–Jensen (LJ), incubation was at 37°C for a maximum of 56 days. Solid cultures with visible contaminants were recorded as contaminated.

Xpert MTB/RIF

One ml of the sputum was added to 2 ml of sample reagent buffer and incubated at room temperature, shaking intermittently to ensure complete sputum lysis. 2 ml of the mucolyzed sample were then used for Xpert testing per the raw sputum procedure in the Xpert package insert. Processed sputa were immediately loaded into individual Xpert cartridges and tested on the Xpert machine. Cycle threshold (CT) values of five probes targeting the *rpoB* gene were recorded. As described elsewhere, the mean CT value of all five probes was used as a measure of bacillary burden (2).

Sputum processing for RNA assays

Sputum collection and laboratory assays were as previously described (3) with slight modifications. Briefly, two spontaneously expectorated sputa were collected into RNA-preservative composed of 5M GTC, 10 mM (Tris(2-carboxyethyl)phosphine) (TCEP); 25 mM sodium citrate, 0.5% N-lauroyl sarcosine, sodium salt; 1.0% Tween-80; 0.02% Antifoam A; 100 mM Tris-HCl; 0.1 mM EDTA; and 23% ethanol. Sputa were homogenized by vortexing with sterile beads and stored at −80°C for 8 months before they were shipped on dry ice to the University of Colorado for subsequent molecular laboratory assays.

Thawed sputum-GTC-TCEP mixture was transferred into two pre labeled sterile 15 ml Falcon tubes leaving the glass beads in the original 50 ml Falcon tube. The 15 ml Falcon tubes containing the sputum-GTC-TCEP mixture were centrifuged for 30 minutes at 3000 RPM. The supernatant was removed leaving approximately 200 μL of the GTC-TCEP with the pellet. Pellets from both 15 ml Falcon tubes were resuspended and combined in one 1.5 ml tube and centrifuged for 3 minutes at 8800 RCF. The resulting supernatant was discarded, and the pellet resuspended by vortexing in 1 ml Trizol before RNA extraction was done.

RNA extraction

Tubes containing sputum/Trizol mixture were homogenized prior to RNA extraction and RNA was extracted using the phenol/chloroform method as previously described [1]. Briefly, 0.4 ml of 0.1 mm silica beads (Matrix B) (MP Biomedicals, #116911050) were added to each tube and the sample was homogenized thrice for 30 seconds at 6M/second on the Fastprep24 bead-beater (MP Biomedicals). After the homogenization, the RNA was extracted with chloroform and centrifuged for 10 minutes at 20800g. The aqueous phase was transferred to a new tube and precipitated overnight a volume of isopropanol equal to the aqueous phase volume with 1:10 volume of 5M ammonium acetate. The RNA was washed twice with 70% ethanol, then purified using the Maxwell RSC simply RNA tissue kit on the Maxwell RSC instrument (Promega) following the protocol described by Promega (Maxwell RSC simply RNA Tissue Kit, Promega). For every batch, a negative extraction control was included to test for cross-contamination between samples, and 2 µl of extraction control to track RNA extraction efficiency (ERCC) was added to each sample (4).

Quantification of rRNA burden via RT-qPCR

rRNA burden was expressed as the absolute count of 16S or 23S rRNA gene transcript copies adjusted for RNA extraction efficiency, dilution factors, and sputum weight. RNA extraction efficiency was tracked using RNA retention percentage. After extraction, RNA was reverse transcribed, and the resulting cDNA was diluted to 1:10. Then, 4 µL of the diluted cDNA was used as input in a RT-qPCR step for the quantification of rRNA burden. Concurrently, two templates in the ERCC mix were targeted by RT-qPCR (ERCC002 and ERCC130), the ERCC primers were at 300 nM final concentration, and the probes were at 100 nM final concentration

(4). RNA retention percentage was calculated by taking the inverse of the fold change between the CT values of the specimen and the control.

Quantification of RS Ratio via droplet digital PCR

RNA was reverse transcribed with SuperScript VILO cDNA synthesis kit (Invitrogen) according to the manufacturer's protocol, except that reverse transcription at 42°C was performed for 120 minutes. Each sample was split into approximately into ~20,000 droplets using either manual droplet generator (QX200, Bio-Rad) or auto droplet generator (AutoQX200, Bio-Rad). Transcript copies were quantified using the QX200 Droplet Digital PCR system (Bio-Rad). The primers/probes sequences and thermocycling conditions have been previously published (4). Reaction were run in duplex, ETS1 with 23S with ddPCR SuperMix for Probes (no dUTP) (Bio-Rad). All primers were 900 nM final concentration and all probes were 250 nM final concentration. The thermocycling conditions for all ddPCR reactions were: initial denaturation at 95°C for 10 minutes, 40 cycles of 94°C for 30 seconds and 60°C for 60 seconds with a 2°C/second ramp rate, and a final hold at 98°C for 10 minutes. RS Ratio estimates were calculated within each duplexed reaction by the QX200 ddPCR system (QuantaSoft software) (Bio-Rad).

SUPPLEMENTAL FIGURES & TABLES

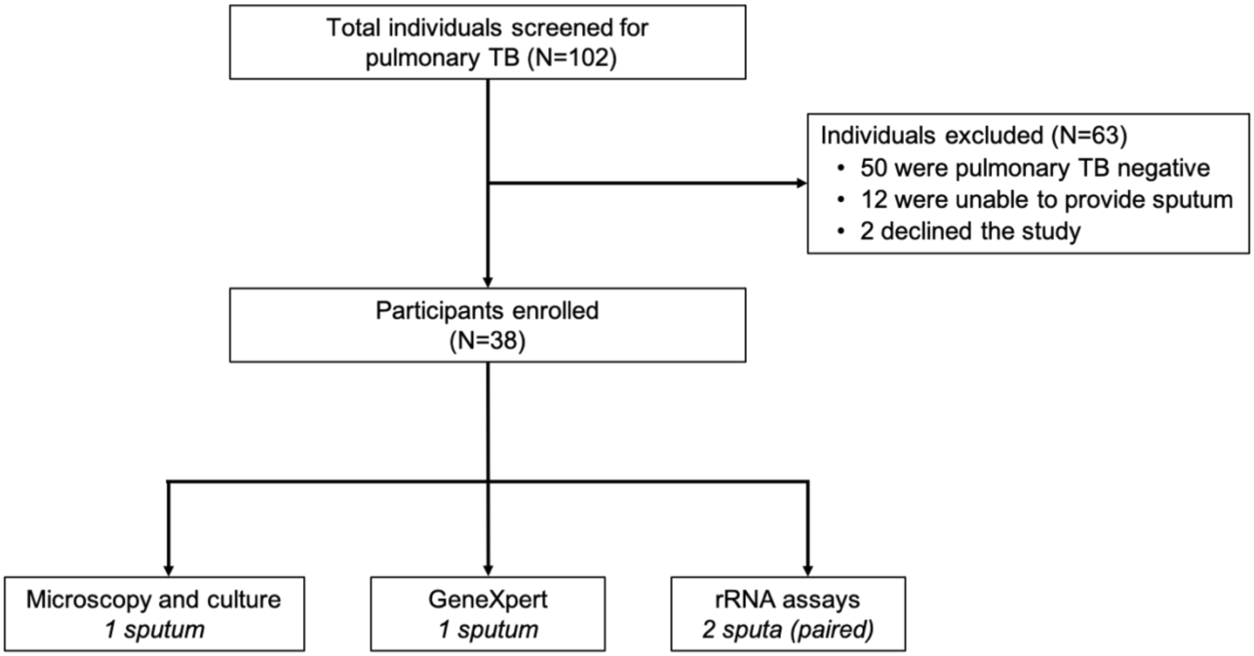

FIG S1. Participant recruitment and sputum collection.
A total of 38 participants with drug-susceptible pulmonary were enrolled. Each patient provided 4 sputum specimens.

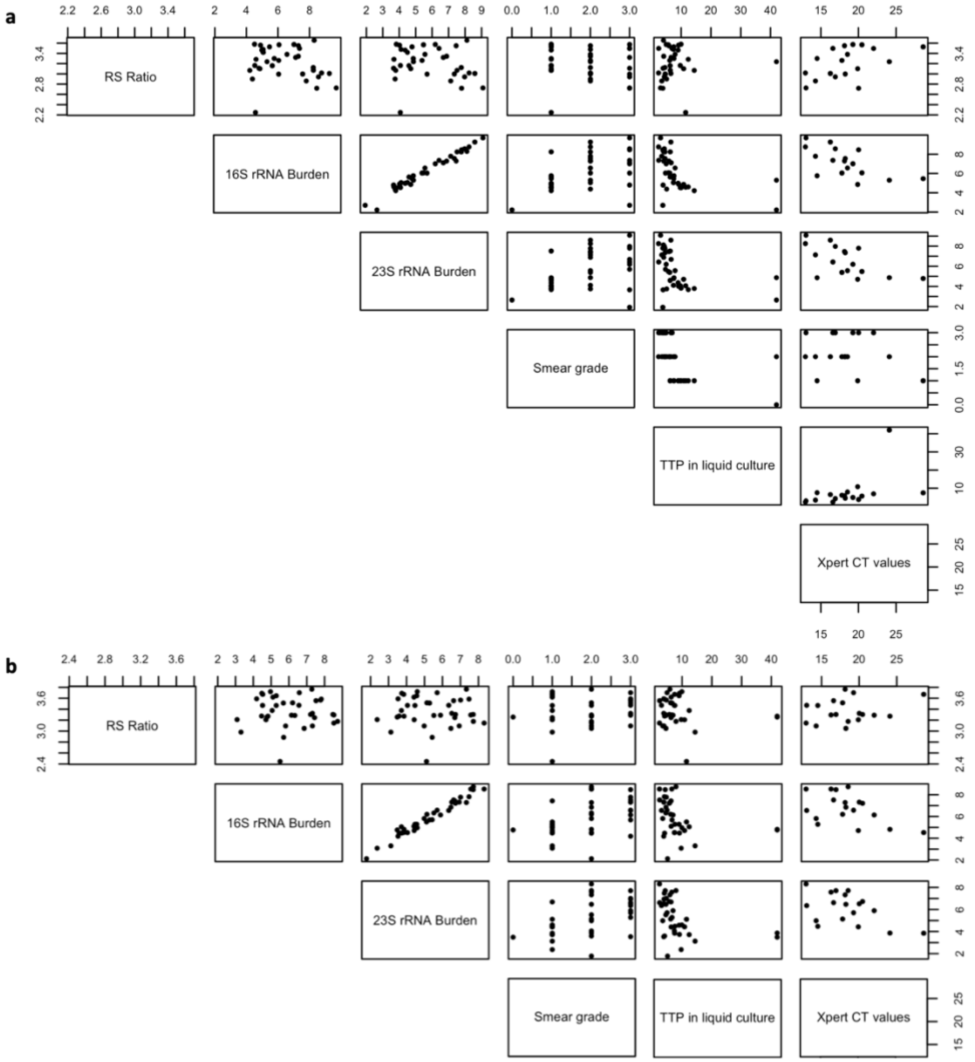

FIG S2. Graphical representation of results presented in Table 3.
Association between the RS Ratio in SS1 (a) or SS2 (b) and markers of *Mtb* burden (16S and 23S rRNA burden, smear grade TTP and Xpert CT value).

TABLE S1. Associations of gender, HIV status and smoking with RS Ratio and markers of *Mtb* burden.

Associations were tested using two-sample Wilcoxon tests. P-values are shown.

	Sex (Male vs. Female)	*HIV status (Negative vs. Positive)*	*Smoking (non-smokers vs. smokers)*
RS Ratio	0.16	0.54	0.62
16S burden	0.67	0.60	0.89
23S burden	0.64	0.76	0.85
Smear grade	0.23	0.32	0.17
TTP in liquid culture	0.56	0.21	0.68
Xpert CT values	0.03	0.57	0.82

REFERENCES

1. Tuberculosis Division, International Union Against Tuberculosis and Lung Disease, Tuberculosis bacteriology—priorities and indications in high prevalence countries: position of the technical staff of the Tuberculosis Division of the International Union Against Tuberculosis and Lung Disease, *Int. Union Tuberc. Lung Dis.* **Volume 9** (2005) 355-361(7).
2. C. F. Hanrahan, G. Theron, J. Bassett, K. Dheda, L. Scott, W. Stevens, I. Sanne and A. Van Rie, Xpert MTB/RIF as a Measure of Sputum Bacillary Burden. Variation by HIV Status and Immunosuppression, *Am. J. Respir. Crit. Care Med.* **189** (2014) 1426–1434.; DOI:10.1164/rccm.201312-2140OC
3. N. D. Walter, B. C. de Jong, B. J. Garcia, G. M. Dolganov, W. Worodria, P. Byanyima, E. Musisi, L. Huang, E. D. Chan, T. T. Van, M. Antonio, A. Ayorinde, M. Kato-Maeda, P. Nahid, A. M. Leung, A. Yen, T. E. Fingerlin, K. Kechris, et al., Adaptation of Mycobacterium tuberculosis to Impaired Host Immunity in HIV-Infected Patients, *J. Infect. Dis.* **214** (2016) 1205–1211.; DOI:10.1093/infdis/jiw364
4. N. D. Walter, S. E. M. Born, G. T. Robertson, M. Reichlen, C. Dide-Agossou, V. A. Ektnitphong, K. Rossmassler, M. E. Ramey, A. A. Bauman, V. Ozols, S. C. Bearrows, G. Schoolnik, G. Dolganov, B. Garcia, E. Musisi, W. Worodria, L. Huang, J. L. Davis, et al., Mycobacterium tuberculosis precursor rRNA as a measure of treatment-shortening activity of drugs and regimens, *Nat. Commun.* **12** (2021) 2899.; DOI:10.1038/s41467-021-22833-6
